# Age‐ and Sex‐Specific Distribution and Reference Values of Coronary Artery Calcium in a Large Asymptomatic Japanese Cohort

**DOI:** 10.1161/JAHA.125.046403

**Published:** 2026-02-12

**Authors:** Hidenobu Takagi, Masaharu Hirano, Takashi Asano, Kensuke Nishimiya, Taku Obara, Hideki Ota, Junichi Taguchi, Kei Takase

**Affiliations:** ^1^ Department of Advanced Radiological Imaging Collaborative Research Tohoku University Sendai Japan; ^2^ Department of Diagnostic Radiology Tohoku University Hospital Sendai Japan; ^3^ Department of Early Imaging Diagnosis Joint Research Tohoku University Sendai Japan; ^4^ Yamanakako Clinic Minamitsuru Japan; ^5^ Department of Cardiovascular Medicine Tohoku University Graduate School of Medicine Sendai Japan; ^6^ Division of Molecular Epidemiology, Graduate School of Medicine Tohoku University Sendai Japan; ^7^ Department of Preventive Medicine and Epidemiology, Tohoku Medical Megabank Organization Tohoku University Sendai Japan; ^8^ Department of Pharmaceutical Sciences Tohoku University Hospital Sendai Japan; ^9^ Medical IT center Tohoku University Hospital Sendai Japan; ^10^ Tokyo Midtown Clinic Minato Tokyo Japan

**Keywords:** atherosclerosis, computed tomography, coronary artery calcium, Race and Ethnicity, Primary Prevention, Computerized Tomography (CT)

## Abstract

**Background:**

The clinical use of coronary artery calcium (CAC) scoring for risk stratification in Japan is limited by the absence of population‐specific reference data, as applying Western‐derived thresholds is inappropriate because of known ethnic variations. This study aimed to establish the first comprehensive, age‐ and sex‐specific CAC reference values for a healthy Japanese population.

**Methods:**

In this single‐center retrospective study, we analyzed data from 4891 asymptomatic Japanese adults (63.2% men; median age, 58 years) without a history of atherosclerotic cardiovascular disease or diabetes. Age‐ and sex‐specific CAC percentile curves were generated using nonparametric regression modeling.

**Results:**

Men exhibited a higher CAC burden than women, with scores increasing with age in both sexes. The relationship between detectable CAC (CAC >0) and age was nonlinear: concave down for men and concave up for women, indicating different progression patterns. Compared with MESA (Multi‐Ethnic Study of Atherosclerosis) data, the Japanese cohort had a markedly lower CAC burden than White participants and systematically lower scores than Chinese American women.

**Conclusions:**

This study provides the first large‐scale, age‐ and sex‐specific CAC reference values for a healthy Japanese population. The generated percentile curves offer a practical tool for clinicians to immediately assess a patient’s CAC burden relative to their peers, underscoring that using foreign‐derived thresholds is inappropriate for risk stratification in Japan.

**Nonstandard Abbreviations and Acronyms**
MESA: Multi‐Ethnic Study of Atherosclerosis

**Clinical Perspective**

**What Is New?**

- This study provides the first large‐scale, contemporary, age‐ and sex‐specific coronary artery calcification percentile reference values derived from over 4800 asymptomatic Japanese adults.
- Our data quantitatively confirm that the Japanese population has a significantly lower coronary artery calcification burden than Western cohorts and exhibits clinically relevant heterogeneity compared to other East Asian populations, such as Chinese Americans.

**What Are the Clinical Implications?**

- These findings underscore that applying Western‐derived or generalized Asian coronary artery calcification thresholds is inappropriate for Japanese patients; our population‐specific percentile curves provide a practical tool for clinicians in Japan to accurately assess an individual’s risk relative to their peers and guide preventive therapy decisions, especially in the large intermediate‐risk group.

Atherosclerotic cardiovascular disease (ASCVD) remains a leading cause of morbidity and mortality worldwide.^1^ Notably, approximately 50% of ASCVD‐related deaths occur in asymptomatic individuals, highlighting the critical need for accurate risk stratification in asymptomatic populations and the timely initiation of preventive interventions in high‐risk individuals.^2^ International guidelines recommend ASCVD risk assessment using prediction models, followed by lifestyle modification and preventive pharmacotherapy based on estimated risk.^3^, ^4^, ^5^ However, conventional risk models frequently overestimate or underestimate ASCVD risk,^6^, ^7^ potentially leading to suboptimal therapeutic guidance. Although efforts have been made to refine existing models and develop novel predictive tools, risk stratification based solely on conventional parameters —such as age, sex, medical history, family history, and laboratory data— has inherent limitations. Additional modalities are needed to enhance risk prediction, particularly to appropriately reclassify individuals categorized as intermediate risk and to facilitate individualized preventive strategies.

Coronary artery calcification (CAC) is a well‐established surrogate marker of subclinical atherosclerosis and provides incremental prognostic value beyond traditional risk factors.^8^ Numerous studies, including large‐scale prospective cohorts, have demonstrated that CAC scoring significantly improves ASCVD risk prediction and enhances the reclassification of individuals with borderline or intermediate risk.^3^, ^9^, ^10^ In recognition of this evidence, the 2019 American College of Cardiology/American Heart Association (ACC/AHA) guidelines provide a Class IIa recommendation for the use of CAC scoring to supplement conventional risk assessment.^3^ In contrast, Japanese guidelines currently recommend risk assessment based solely on conventional factors and do not endorse CAC scoring for primary prevention, citing concerns about ethnic differences.^5^

The importance of population‐specific reference values is well‐documented. MESA (Multi‐Ethnic Study of Atherosclerosis) revealed substantial variations in CAC burden by ethnicity,^11^ and significant efforts have been made to establish reference values for various populations. Notably, a large‐scale study in over 31 000 asymptomatic Koreans has provided robust data for that population,^12^ and a recent meta‐analysis, including data from Japan, has summarized CAC reference ranges across multiple ethnicities.^13^ However, despite these crucial contributions, the direct application of such data to the Japanese population may be limited because of potential differences in lifestyle, diet, and genetic background, even within East Asian groups. Furthermore, existing data often lack the detailed, continuous percentile distributions necessary for a practical clinical tool that allows for the nuanced risk assessment of an individual patient relative to their peers. To date, a large‐scale, contemporary reference standard tailored specifically for the Japanese population remains undefined.

Therefore, this study aimed to define the age‐ and sex‐specific distribution of CAC in a large cohort of healthy Japanese individuals and to develop a practical reference tool with detailed percentile curves to support individualized ASCVD risk assessment and prevention in Japan.

## METHODS

### Data Availability Statement

The data that support the findings of this study are not publicly available because of ethical restrictions protecting patient privacy. De‐identified data underlying the results presented in this article (including tables, figures, and supplemental material) may be made available from the corresponding author upon reasonable request. Any request will require a formal data use agreement and approval from the Institutional Review Board of Tohoku University Graduate School of Medicine before data can be shared.

### Study Design and Cohort

This single‐center retrospective observational study was approved by the Ethics Committee of Tohoku University Graduate School of Medicine, Sendai, Japan (identifier: 2023‐1‐855). The requirement for written informed consent was waived because of the retrospective nature of the study. Asymptomatic individuals who underwent comprehensive health check‐ups at Yamanakako Clinic (Yamanashi, Japan) between 2016 and 2023 were included. CAC scanning was performed as an optional, self‐paid component of this comprehensive health check‐up program. For individuals with multiple CAC examinations, only the earliest result was used. Exclusion criteria were the absence of CAC scanning (representing individuals who did not select this optional scan) or a history of percutaneous coronary intervention, myocardial infarction, stroke or transient ischemic attack, heart failure, or diabetes.

### Clinical Data Collection and Laboratory Measurements

Data on age, sex, medical history, antihypertensive and lipid‐lowering medication use, and smoking status were obtained through a standardized questionnaire and verified using electronic medical records. Smoking status was categorized as “Never” (never smoked), “Past” (former smokers who had quit), or “Current” (actively smoking at the time of assessment). Systolic and diastolic blood pressure were measured using an automated sphygmomanometer following at least 3 minutes of rest in a seated position. Blood samples were collected after an overnight fast, and serum lipid profiles (total cholesterol, low‐density lipoprotein cholesterol, high‐density lipoprotein cholesterol, triglycerides) and hemoglobin A1c levels were analyzed using standardized enzymatic methods. For the purposes of risk factor categorization and comparison with established cohorts,^14^ obesity was defined as a body mass index ≥25 kg/m^2^. Hypertension was defined as a systolic blood pressure≥140 mm Hg, a diastolic blood pressure≥90 mm Hg, or current use of antihypertensive medication. Hypercholesterolemia was defined as a total cholesterol level≥5.7 mmol/L (220 mg/dL). These definitions were chosen to align with those used in the Hisayama Study.^14^

### Risk Stratification

The 10‐year ASCVD risk was calculated for each participant using a validated risk prediction model derived from the Hisayama Study.^15^ Risk categories were defined as low risk (<5.0%), intermediate risk (5.0% to 19.9%), and high risk (≥20%), consistent with the American guideline.^3^

### CAC Scanning and Measurements

CAC scanning was performed using a standardized protocol with a 64‐slice computed tomography scanner (Biograph mCT Flow, Siemens Healthineers, Erlangen, Germany) without contrast and using electrocardiography‐gated acquisition. CAC was quantified by experienced technicians using semiautomated software (AZE Virtual Place; AZE, Inc, Tokyo, Japan) according to the Agatston method.^16^ The analysis software was updated during the study period, with the Raijin module used until 2022 and the Class R module from 2023 onwards. CAC scores were recorded in the electronic medical record and categorized as follows: 0 (none), 1 to 10 (minimal), 11 to 100 (mild), 101 to 300 (moderate), 301 to 999 (severe), or >1000 (extensive).^17^

### Statistical Analysis

Continuous variables are presented as medians with interquartile ranges (25th–75th percentiles), and categorical variables as counts and percentages. The distribution of CAC scores in this cohort was highly right skewed for both men and women, as illustrated in Figure S1a. To address this skewness, CAC was log‐transformed as *log*(*CAC* + 1) for all analyses involving continuous CAC values. The distribution of log‐transformed CAC is shown in Figure S1b. Following the methodology used in the MESA study for establishing CAC percentiles,^18^ we first modeled the probability of detectable CAC (CAC >0) as a function of age, stratified by sex, using nonparametric locally weighted scatterplot smoothing regression to account for the highly skewed distribution of CAC in asymptomatic populations. Next, we modeled the mean of *log*(*CAC* + 1) as a function of age separately for each sex, using a smoothing span of 0.7. Residuals from this model were pooled, ranked, and used to calculate the 1st through 100th percentiles of the residual distribution. Finally, these percentiles were added to the fitted mean estimates to derive age‐ and sex‐specific percentile values for CAC. All analyses were performed using R version 4.4.0.^19^

## RESULTS

### Participant Characteristics

A total of 6843 individuals underwent health check‐ups between 2016 and 2023 and were screened for study eligibility. Of these, 1097 individuals who did not undergo CAC testing and 141 individuals with coronary stents were excluded. Among the remaining 5605 individuals, those with a history of myocardial infarction (n=37), stroke or transient ischemic attack (n=172), heart failure (n=13), or diabetes (n=495) were excluded. Consequently, 4891 individuals were included in the final analysis. The characteristics of the study cohort are summarized in Table 1. The cohort was predominantly male (n=3094; 63.2%), with a median age of 58 years (interquartile range, 51–67). The most common age category among men was 45 to 54 years (n=1032; 33.3%), whereas among women, the 55 to 64 years age category was most prevalent (n=669; 37.2%). The age‐ and sex‐specific distributions of these risk scores are presented in Table S1. The estimated risk increased with age in both sexes.

**Table 1:** Characteristics of the Study Population

Variables	Overall (N = 4891)	Men (N = 3094)	Women (N = 1797)
Age, y	58 (51–67)	56 (48–65)	62 (55–69)
Age category			
35–44 y	440 (9.0%)	400 (13%)	40 (2.2%)
45–54 y	1389 (28%)	1032 (33%)	357 (20%)
55–64 y	1529 (31%)	860 (28%)	669 (37%)
65–74 y	1166 (24%)	611 (20%)	555 (31%)
≥75 y	367 (7.5%)	191 (6.2%)	176 (9.8%)
Body mass index, kg/m^2^	23.7 (21.5–25.9)	24.5 (22.6–26.6)	21.8 (19.9–24.4)
Obesity, n (%)	1674 (34%)	1317 (43%)	357 (20%)
Hypertension, n (%)	2091 (43%)	1414 (46%)	677 (38%)
Systolic BP, mm Hg	128 (117–138)	129 (118–138)	127 (115–138)
Diastolic BP, mm Hg	80 (72–87)	81 (74–88)	76 (69–83)
Systolic BP in hypertensive individuals, mm Hg	140 (130–148)	139 (129–148)	142 (133–149)
Diastolic BP in hypertensive individuals, mm Hg	86 (78–93)	88 (79–94)	83 (75–90)
Hypercholesteremia, n (%)	2447 (50%)	1410 (46%)	1037 (58%)
Serum total cholesterol, mmol/L	5.7 (5.0–6.3)	5.5 (5.0–6.2)	5.8 (5.2–6.5)
Serum LDL cholesterol, mmol/L	3.4 (2.8–3.9)	3.3 (2.8–3.9)	3.4 (2.9–4.0)
Serum HDL cholesterol, mmol/L	1.6 (1.3–2.0)	1.5 (1.3–1.8)	1.9 (1.6–2.2)
Serum triglycerides, mmol/L	0.71 (0.59–0.86)	0.65 (0.55–0.78)	0.82 (0.70–0.97)
Hemoglobin A1c, %	5.1 (4.9–5.4)	5.1 (4.9–5.4)	5.1 (5.0–5.4)
Smoking status			
Never	2207 (45%)	845 (27%)	1362 (76%)
Past	1538 (31%)	1254 (41%)	284 (16%)
Current	1145 (23%)	994 (32%)	151 (8.4%)
Antihypertensive medications	1153 (24%)	764 (25%)	389 (22%)
Lipid‐lowering medications			
Statin	641 (13%)	331 (11%)	310 (17%)
Non statin	195 (4.0%)	136 (4.4%)	59 (3.3%)
Anti‐platelet agents	104 (2.1%)	78 (2.5%)	26 (1.4%)
10‐y ASCVD risk			
Low (<5.0%)	1257 (26%)	599 (19%)	658 (37%)
Intermediate (5.0%–19.9%)	3126 (64%)	2059 (67%)	1067 (59%)
High (≥20%)	508 (10%)	436 (14%)	72 (4.0%)
CAC group			
0	2977 (61%)	1691 (55%)	1286 (72%)
1–10	476 (9.7%)	336 (11%)	140 (7.8%)
11–100	767 (16%)	554 (18%)	213 (12%)
101–300	374 (7.6%)	270 (8.7%)	104 (5.8%)
301–999	225 (4.6%)	179 (5.8%)	46 (2.6%)
≥1000	72 (1.5%)	64 (2.1%)	8 (0.4%)

Continuous variables are summarized as median and interquartile ranges (25%–75%) in parentheses, and categorical variables are summarized raw number and percentages in parentheses. BP indicates blood pressure; LDL, low‐density lipoprotein; HDL, high‐density lipoprotein; and CAC, coronary artery calcium.

### Prevalence of CAC by Age and Sex

**Figure 1:**
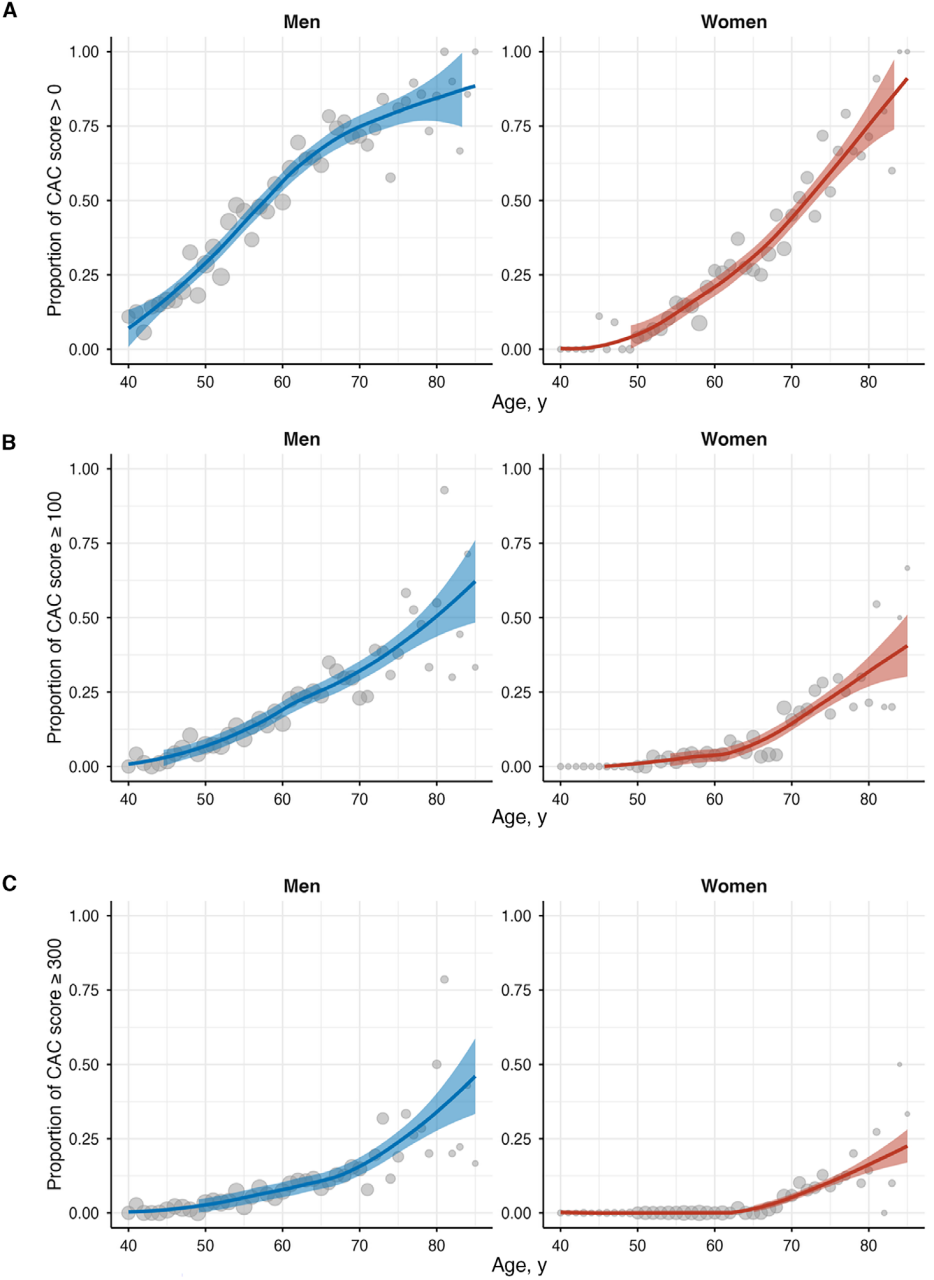
Estimated and observed probability of coronary artery calcium by age and sex. The graphs show the probability of (**A**) detectable CAC (score>0), (**B**) moderate or higher CAC (score≥100), and (**C**) severe or higher CAC (score≥300). Solid lines indicate the probability estimated by locally weighted scatterplot smoothing (LOWESS) regression. Shaded areas represent 95% CIs. Dots represent the observed prevalence within 5‐year age intervals. CAC indicates coronary artery calcium.

The estimated and observed prevalence of detectable coronary artery calcium (CAC score>0), mild to extensive CAC (CAC score≥100), and severe to extensive CAC (CAC score≥300) by age and sex are shown in Figure 1. In both men and women, the probability of detectable CAC increased with advancing age. The estimated probability curve for detectable CAC showed an upward convex shape in men, with a steep increase after approximately 40 years of age. In women, the curve was downward convex with a gradual increase observed after approximately 55 years of age. Among men, the estimated prevalence of CAC >0 exceeded 15% from approximately 45 years of age and surpassed 25% at approximately 50 years of age, with a progressive increase thereafter. In contrast, women exhibited a lower prevalence of detectable CAC across all age groups, with rates exceeding 15% around 55 years of age and surpassing 25% at approximately 60 years.

Similarly, the prevalence of CAC ≥100 (Figure 1B) and CAC ≥300 (Figure 1C) increased with age in both sexes, with men consistently showing higher rates than women. For CAC ≥100, the estimated prevalence exceeded 15% in men from approximately 60 years of age, whereas in women, it remained <15% until approximately 75 years of age. For CAC ≥300, the prevalence exceeded 15% in men after approximately 70 years, while in women, the prevalence surpassed 15% only after approximately 80 years of age.

### Distribution of CAC by Age and Sex

**Figure 2:**
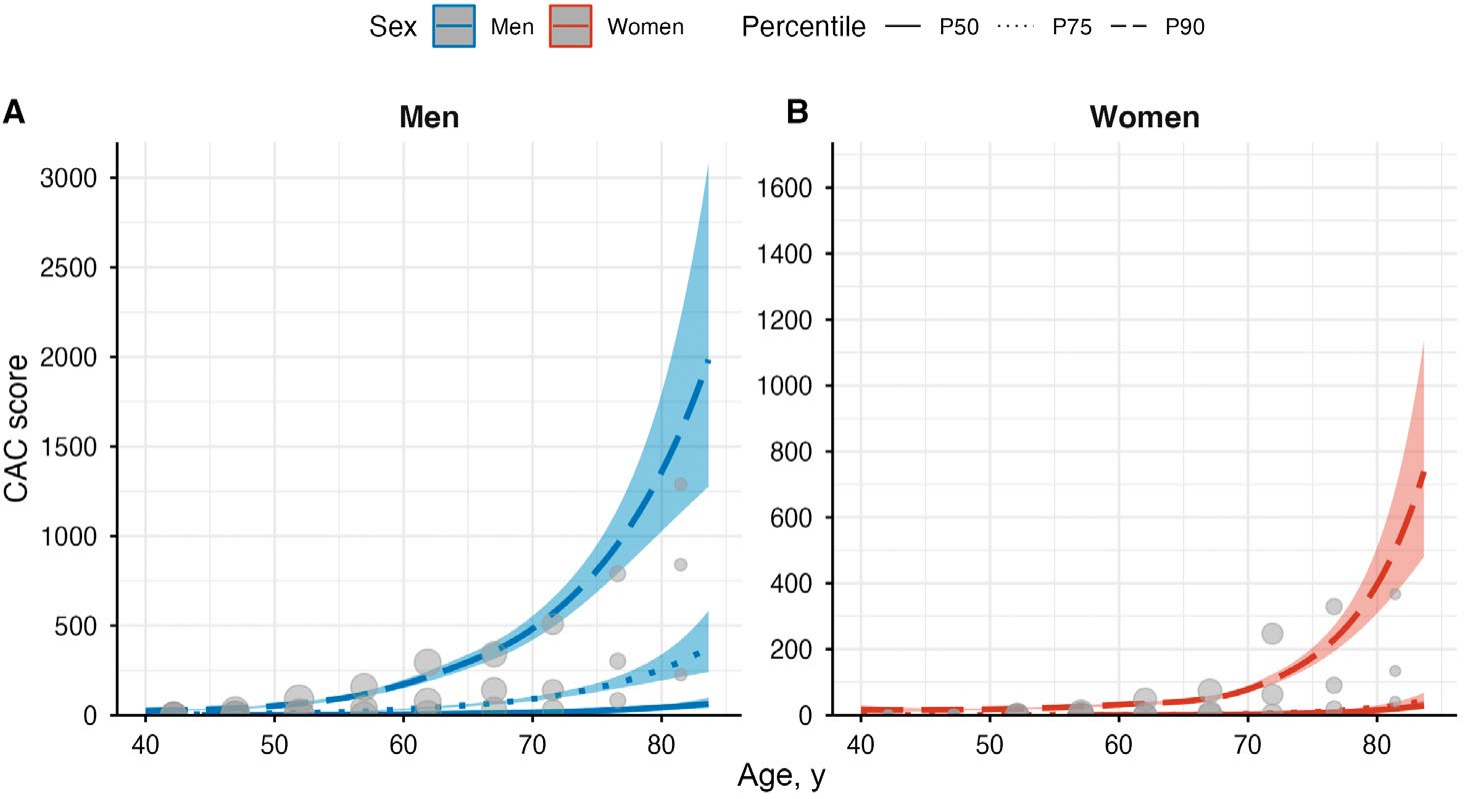
Estimated percentiles of coronary artery calcium score by age and sex. Each plot shows the estimated curves for the 50th, 75th, and 90th percentiles of the CAC score distribution across age for men (**A**) and women (**B**). The observed empirical percentiles for each 5‐year age interval are overlaid as dots for reference. CAC indicates coronary artery calcium.

The estimated age‐ and sex‐specific percentiles of CAC are shown in Figure 2 and summarized numerically in Table 2. In both men and women, each estimated percentile of CAC increased exponentially with age. The increase was more pronounced in men, with higher CAC values observed across all age groups compared with women. Among men, the 75th percentile of CAC was >0 in the 45 to 54 age group, reaching 55 in the 55 to 64 group, 140 in the 65 to 74 group, and 441 in the ≥75 years group. In women, the 75th percentile remained 0 in the 55 to 64 age group, increasing to 20 in the 65 to 74 group and 137 in the ≥75‐year age group.

**Table 2:** Estimated Percentile of CAC Score by Age Category and Sex

	Age 35–44 y	Age 45–54 y	Age 55–64 y	Age 65–74 y	Age ≥75 y
Men					
25th percentile	0	0	0	0	7
50th percentile	0	0	2	29	106
75th percentile	0	2	55	140	441
90th percentile	1	58	237	407	1138
95th percentile	14	151	430	823	1641
Women					
25th percentile	0	0	0	0	0
50th percentile	0	0	0	0	31
75th percentile	0	0	0	20	137
90th percentile	0	0	23	131	410
95th percentile	0	2	81	259	711

CAC indicates coronary artery calcium.

## DISCUSSIONS

In this large‐scale study of asymptomatic Japanese individuals, we characterized the age‐ and sex‐specific distribution of CAC scores. To our knowledge, this study represents one of the largest analyses of CAC in a healthy Japanese population and provides reference percentiles to guide clinical interpretation. Our main findings are 2‐fold. First, the prevalence of detectable CAC and CAC scores increased steadily with age in both sexes. Compared with women, men exhibited an earlier onset and higher burden of CAC across all age groups, indicating more accelerated subclinical atherosclerosis. Second, when compared with data from MESA, Japanese individuals demonstrated consistently lower CAC scores than their White counterparts across all age and sex groups.

Aging promotes vascular calcification through multiple pathways, including endothelial dysfunction, oxidative stress, chronic low‐grade inflammation, arterial remodeling, and osteogenic transdifferentiation of vascular smooth muscle cells.^20^, ^21^, ^22^, ^23^ In our cohort, men exceeded a 15% prevalence of detectable CAC (CAC >0) by age 45 years, whereas women reached this threshold approximately a decade later. A similar lag was observed for moderate (CAC ≥100) and severe (CAC ≥300) calcification, suggesting delayed vascular aging in women. These patterns are concordant with findings from MESA and other large cohorts,^18^, ^24^, ^25^, ^26^, ^27^ which consistently report an earlier onset and more rapid progression of calcification in men. The vasoprotective effects of estrogen are widely proposed to underlie these sex differences,^28^ with epidemiological data showing a sharp rise in coronary artery disease incidence in women following menopause. While the fundamental relationships between advancing age, male sex, and increased CAC appear universal across ethnicities, our findings underscore the need for Japanese‐specific reference percentiles to account for systematically lower CAC scores relative to thresholds derived from Western cohorts.

**Figure 3:**
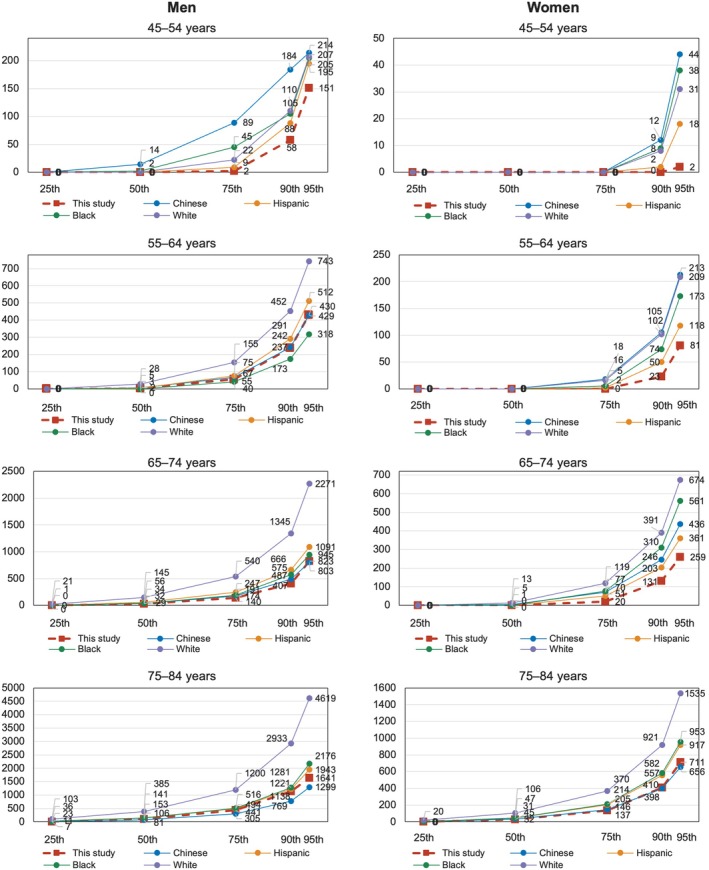
Comparison of median coronary artery calcium scores among different ethnic groups. The CAC scores for Japanese men and women from this study are compared with those of White, Black, Hispanic, and Chinese American participants from the MESA (Multi‐Ethnic Study of Atherosclerosis).^18^ This figure highlights the differences in CAC burden across ethnicities. CAC indicates coronary artery calcium.

Our study contributes to the growing body of literature defining CAC distributions in East Asian populations. A large‐scale study in over 31 000 asymptomatic Koreans has provided robust reference data,^12^ and a recent meta‐analysis summarized CAC ranges across multiple ethnicities, including some from Japan. Our results are broadly consistent with these studies, confirming a lower CAC burden in East Asians compared with Western populations. However, we also observed significant heterogeneity within Asian subgroups. As illustrated in Figure 3, which situates our cohort within the context of MESA’s multi‐ethnic data, Japanese women in our cohort exhibited systematically lower CAC scores than Chinese American women in MESA, a finding that highlights potential differences in lifestyle, diet, or genetic factors that are not captured by broad ethnic categories. This observation is further supported by studies like MASALA, which found that South Asians have a CAC burden comparable to or higher than White Americans.^29^ These findings reinforce that “Asian” is not a monolithic category for ASCVD risk. Our results also align with previous, smaller‐scale Japanese studies, such as the Shiga Epidemiological Study, which noted a lower CAC burden in Japanese men that diverged from US White men with age.^30^ The unique contribution of our study is the provision of detailed, continuous percentile distributions from a large, contemporary Japanese cohort. This offers a more granular and clinically applicable tool for individual risk assessment than was previously available. By allowing clinicians to place a patient’s CAC score in the context of their direct peers, our reference curves strengthen the call for population‐specific thresholds to avoid the potential misclassification of risk that may arise from applying foreign‐derived or generalized Asian data.

To validate our cohort as an appropriate source for contemporary Japanese reference values, we compared its baseline characteristics and estimated ASCVD risk profile to established cohorts. As detailed in Table S2, our cohort (baseline 2016–2023) exhibited differences compared with the Hisayama Study cohort (baseline 2002).^14^ Specifically, our male participants had a higher prevalence of obesity (43% versus 29%) and hypercholesterolemia (46% versus 22%). Women in our cohort also had a higher prevalence of hypercholesterolemia (58% versus 35%). Furthermore, while the prevalence of diagnosed hypertension was higher in our cohort (men: 46% versus 41%; women: 38% versus 30%), as was the use of antihypertensive medications (men: 25% versus 17%; women: 22% versus 16%), the current smoking rate among men was lower (32% versus 47%). These differences are consistent with the temporal trends reported by Hata et al. themselves, which demonstrated an increasing prevalence of obesity, hypercholesterolemia, and hypertension, alongside decreasing smoking rates within the Japanese population over time.^14^ Therefore, the discrepancies between our cohort and their 2002 baseline data likely reflect this well‐documented shift in the cardiovascular risk profile of the Japanese population over the last two decades, underscoring the necessity of establishing updated, contemporary reference values. Furthermore, the ASCVD risk distribution calculated for our cohort reveals a substantial proportion of individuals classified into the intermediate‐risk category (5.0%–19.9%), particularly in men aged 45 and older (73% in 45–54 years) and women aged 55 and older (57% in 55–64 years). This finding is significant as it identifies the specific, large patient population in modern Japan that, according to US guidelines,^3^ stands to benefit most from further risk reclassification using the CAC percentile curves we have presented.

This study has several limitations. First, the single‐center design, using data from a health check‐up facility, may limit the generalizability of our findings to the entire Japanese population. Participants in such programs may be more health‐conscious or have a different socioeconomic status compared with the general public, potentially introducing selection bias. Second, the study’s cross‐sectional nature precludes the assessment of CAC progression over time or the prognostic value of these CAC distributions for predicting future ASCVD events. We have established reference values, but their ability to predict outcomes in a Japanese context needs validation in prospective cohort studies. Third, although data were collected systematically, the retrospective design relies on the accuracy of existing electronic medical records and self‐reported questionnaires, which may be subject to information bias. Fourth, our study used the Agatston score, which quantifies the established burden of macrocalcification, but not the activity of the underlying biological process. Future research using advanced molecular imaging modalities, such as 18F‐Sodium Fluoride ‐ Positron Emission Tomography ‐ Computed Tomography, may provide deeper insights into the active processes of early‐stage microcalcification in this population.^31^ Finally, while our cohort was ethnically Japanese, the findings may not apply to other ethnic minority groups residing in Japan. Despite these limitations, this study provides the largest and most robust data set to date for CAC reference values in a healthy Japanese population.

In conclusion, this study established comprehensive age‐ and sex‐specific reference values for CAC in a large cohort of asymptomatic Japanese individuals. Our data reveal a substantially lower CAC burden in the Japanese population compared with Western cohorts and highlight key differences even within East Asian subgroups, particularly for women. These findings underscore the inadequacy of applying foreign‐derived CAC thresholds to Japanese patients and provide an essential, population‐specific tool to improve the accuracy of ASCVD risk stratification in Japan. Future prospective studies are warranted to validate the prognostic significance of these Japanese‐specific CAC percentiles for predicting long‐term cardiovascular outcomes.

## Sources of Funding

This work was supported by a Grant‐in‐Aid for Young Scientists from the Japan Society for the Promotion of Science (JSPS KAKENHI Grant Number 23K15150).

## Disclosures

Dr Takagi receives research grant from Canon Medical Systems. The remaining authors have no disclosures to report.

## Supporting information

Tables S1–S2
Figure S1
