# Supplementary material for: Age‐ and Sex‐Specific Distribution and Reference Values of Coronary Artery Calcium in a Large Asymptomatic Japanese Cohort
Source: J Am Heart Assoc. 2026 Feb 12;15(5):e046403. doi: 10.1161/JAHA.125.046403 (PMC13055732; doi:10.1161/JAHA.125.046403)
Supplement: Supplementary file 1 — Tables S1–S2 Figure S1 [file JAH3-15-e046403-s001.pdf]

Supplemental Material

Table S1. Distribution of ASCVD Risk Score across Age and Sex.

Age category	N	Mean ± SD	Median (IQR)	Percentage of Intermediate risk category	Percentage of High risk category
**Men**					
35–44 yrs.	400	3.9 ± 1.2	3.2 (3.2–4.2)	18%	0%
45–54 yrs.	1032	6.3 ± 2.2	5.6 (4.2–7.4)	73%	0%
55–64 yrs.	860	10.9 ± 3.5	9.8 (7.4–12.8)	97%	2%
65–74 yrs.	611	18.0 ± 5.2	16.7 (12.8–21.7)	62%	37%
≥75 yrs.	191	26.8 ± 4.1	27.8 (21.7–30.1)	3%	96%
**Women**					
35–44 yrs.	40	1.7 ± 0.4	1.8 (1.4–1.8)	0%	0%
45–54 yrs.	357	3.3 ± 1.0	3.2 (2.4–4.2)	5%	0%
55–64 yrs.	669	5.5 ± 1.8	5.6 (4.2–7.4)	57%	0%
65–74 yrs.	555	10.3 ± 3.3	9.8 (7.4–12.8)	98%	1%
≥75 yrs.	176	18.2 ± 5.3	16.7 (12.8–21.7)	64%	35%

Data are presented as mean ± standard deviation, median (interquartile range), or percentage. ASCVD risk categories were defined as Intermediate (10-year risk 5.0% to 19.9%) and High (10-year risk ≥20%), consistent with American guidelines.

Table S2. Comparison of Baseline Characteristics Between This Study and the Hisayama Study Cohort

	**This study Men** (n = 3,094)	**Hisayama 2002 Men** (n = 3,108)	**This study Women** (n = 1,797)	**Hisayama 2002 Women** (n = 1,803)
Age, yrs.	57 ± 11	61 ± 12	62 ± 9	62 ± 13
Body mass index, kg/m^2	24.8 ± 3.3	23.4 ± 2.9	22.3 ± 3.4	22.9 ± 3.5
Obesity, %	43%	29%	20%	23%
Hypertension, %	46%	41%	38%	30%
Antihypertensive medications, %	25%	17%	22%	16%
Systolic blood pressure mm Hg	129 ± 16	133 ± 20	127 ± 17	129 ± 20
Diastolic blood pressure, mm Hg	82 ± 11	81 ± 11	76 ± 11	76 ± 12
Systolic BP in hypertensive individuals, mmHg	139 ± 15	148 ± 18	141 ± 14	149 ± 19
Diastolic BP in hypertensive individuals, mmHg	87 ± 12	89 ± 10	82 ± 11	86 ± 11
Hypercholesterolemia, %	46%	22%	58%	35%
Total cholesterol, mmol/L	5.6 ± 0.9	5.1 ± 0.9	5.8 ± 0.9	5.4 ± 0.9
Current smoker, %	32%	47%	8%	8%

Data are presented as mean ± standard deviation or %. Data for the Hisayama Study cohort are from Hata J et al. (Circulation. 2013;128:1198-1205), representing a baseline population survey conducted in 2002.

Figure S1. Distribution of Coronary Artery Calcium (CAC) Scores by Sex.

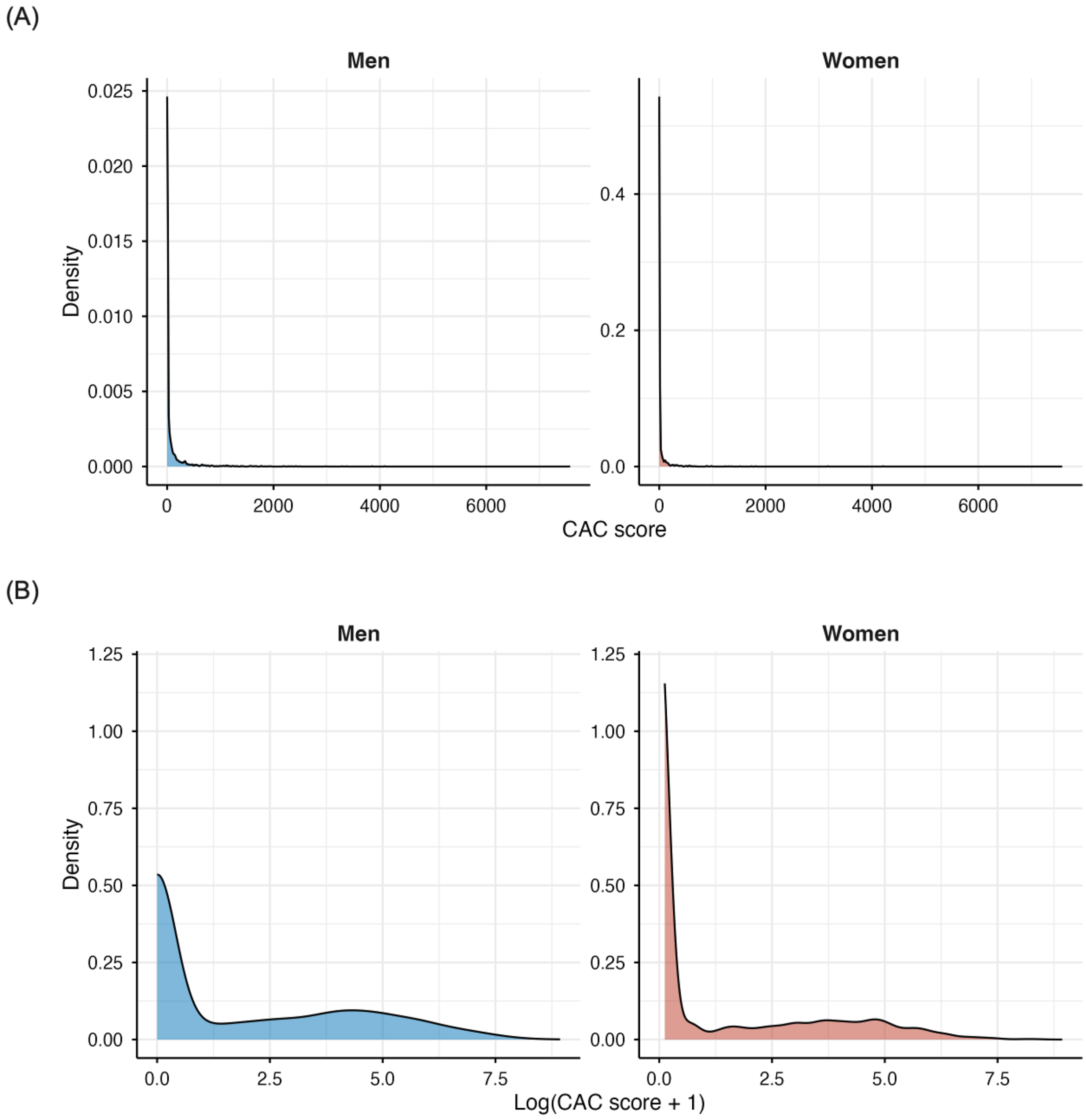

(A) Density plots of the raw Agatston CAC scores for men (**left**) and women (**right**). These plots demonstrate a highly right-skewed distribution, with a large majority of participants having a CAC score of zero.

(B) Density plots of the log-transformed CAC scores, calculated as *log(*CAC+1), for men (**left**) and women (**right**). This transformation yields a more symmetrical distribution, which was used for the subsequent regression modeling to derive the percentile estimates.
